# Do we need to wait? Does reducing time to prostate MRI postbiopsy interfere with staging?

**DOI:** 10.1186/1470-7330-15-S1-P46

**Published:** 2015-10-02

**Authors:** J Rowlands

**Affiliations:** 1 The Shrewsbury and Telford NHS Trust, UK

## Aims

With an MRI scanner at the limit of capacity (and no immediate prospect of an additional scanner) we are unable to offer pre biopsy MRI due to the workload expansion - further 24 slots/ month. We aimed to evaluate if reducing the time between biopsy and scan led to a decrease in staging accuracy due to post biopsy haemorrhage.

## Methods

Retrospective study comparing haemorrhagic artefact and staging accuracy in MRI studies performed before and after a pathway change reduced the time to MRI post biopsy.

## Results

No difference in rate of post biopsy haemorrhage deemed to affect diagnostic accuracy.

No significant staging error in either group.

Reduced time to discussion on MDT led to improvement in treatment time and no RTT (Referral To Treatment) pathway breaches.

Pathway improved by 8.65 days (14% of RTT time).

## Conclusion

In a capacity limited service there is no option to go to prebiopsy service with the increase in demand that would ensue. It is reassuring to know that reducing the time between biopsy and scan results in no difference in number of studies affected by haemorrhage, no difference in diagnostic accuracy and leads to improvement in patient treatment pathway.

